# Enhancing flu vaccine responses in older adults: preliminary insights from the ISOLDA study on immunosenescence and antioxidant and anti-inflammatory approaches

**DOI:** 10.1186/s12979-025-00506-y

**Published:** 2025-03-26

**Authors:** Anna Aiello, Anna Calabrò, Mattia Emanuela Ligotti, Giulia Accardi, Mojtaba Shekarkar Azgomi, Nadia Caccamo, Calogero Caruso, Francesco Dieli, Marco Pio La Manna, Antonio Procopio, Giuseppina Candore

**Affiliations:** 1 Laboratory of Immunopathology and Immunosenescence, Department of Biomedicine, Neurosciences and Advanced Diagnostics, University of Palermo, 90127 Palermo, Italy; 2 Department of Research, Mediterranean Institute for Transplantation and Advanced Specialized Therapies (IRCCS-ISMETT), 90127 Palermo, Italy; 3 Department of Biomedicine, Neuroscience and Advanced Diagnosis, University of Palermo, 90127 Palermo, Italy; 4 Central Laboratory of Advanced Diagnosis and Biomedical Research (CLADIBIOR), AOUP Paolo Giaccone, 90127 Palermo, Italy; 5 Department of Health Sciences, University Magna Graecia of Catanzaro, Viale Europa - Campus Universitario S. Venuta - Loc. Germaneto, 88100 Cosenza, Italy

**Keywords:** Immunosenescence, Inflamm-aging, Influenza, Older adults, Oleuropein, Vaccine

## Abstract

Aging is frequently characterized by an inadequate primary vaccine response, likely due to immunosenescence and inflamm-aging, a low-level, chronic inflammatory state. Both aspects increase the susceptibility of older adults to viral and bacterial infections, resulting in a higher frequency and severity of infectious diseases. In this preliminary study, a cohort of 52 individuals was recruited and divided into two groups: young (age range 21–35) and older adults (> 60 years old). Peripheral blood mononuclear cells (PBMCs) were collected before (time 0, T0) and after (time 1, T1) the immunization with a tetravalent influenza vaccine. Then, T cell immunophenotyping analysis was conducted to investigate how aging and influenza vaccination influence T cell responses. Additionally, the anti-inflammatory and antioxidant effects of oleuropein (OLE), a secoiridoid extracted from extra virgin olive oil, alone or in combination with BIRB 796, a potent inhibitor of p38 MAPK, were explored to enhancing the impact of influenza virus on T cell activation, aiming to identify potential alternatives or complementary strategies to improve traditional flu-vaccine formulations. Statistically significant observations were noted for a decrement in CD8 + T naïve and an increase of effector memory between the young and older adults after flu-vaccination. Moreover, preliminary findings indicate anti-inflammatory and antioxidant properties of OLE and BIRB 796 on T cell responses, particularly regarding Reactive Oxygen Species/Reactive Nitrogen Species modulation, with a trend toward the decrease of pro-inflammatory cytokines (i.e., Interferon-γ (INF-γ), Tumor Necrosis Factor-α (TNF-α)), αalthough without statistical significance.

**Supplementary Information:**

The online version contains supplementary material available at 10.1186/s12979-025-00506-y.

## Introduction

Flu-vaccination is currently the major strategy to prevent infection and the severe outcomes of influenza in high-risk populations, including older adults, i.e., people who are 65 years and older [1]. The risks of hospitalization for influenza in the older population are significantly increased during influenza seasons, with an elevated number of deaths for respiratory disorders-influenza correlated. In the United States, it is estimated that 70–85% of these deaths are older adults [1]. In Europe, seasonal influenza affected 10 to 30% of the population, causing hundreds of thousands of hospitalizations for influenza-related complications, the most among children, oldest and most fragile people [2]. However, while it is proven that influenza vaccination reduces the severity of illness in older adults, its overall effectiveness remains controversial due to the high possibility of infection after vaccination and the exacerbated inflammatory response to the vaccination itself. Indeed, on one hand, several observational studies have shown that the influenza vaccine effectively reduces hospitalization for influenza and decreases the mortality rate but, on the other hand, other studies have noted that the vaccine’s effectiveness is closely tied to the match between the vaccine strains and the circulating virus strains [3–5]. Additionally, the efficacy of the influenza vaccine is lower in individuals aged 65 and older compared to younger adults, diminishing to 30–50% with respect to immunocompetent adults [6, 7].

The questionable results on this reduced influenza vaccination efficiency in older people are probably linked to the immunosenescence hallmarks, i.e., the decrease of naïve cells, the increase of memory cells, and inflamm-aging, i.e., the chronic, low-grade inflammation that develops with advanced age and may contribute to clinical manifestations of several age-related pathologies [8]. So, it is possible to speculate that immunosenescence and infla-mmaging increase the susceptibility to infectious diseases and their associated complications, and negatively affect the effectiveness of vaccination, contributing to lower protection provided by current vaccines in older adults [8].

In this scenario, identifying strategies that could reduce the inflammatory process exacerbated by the vaccination process and mitigate the effects of immunosenescence to improve the older response to flu-vaccine, becomes a challenge.

In recent years, research on natural compounds that could act as adjuvants in vaccine production is growing and, in particular, phytochemicals derived from extra virgin olive oil are identified as inhibitors of inflamm-aging [9, 10]. Phenolic compounds of olive oil include ca. 30 molecules, some with strong antioxidant and anti-inflammatory properties, that could counteract the pathophysiology of age-related diseases, with a relevant role in many anti-aging strategies. Their mechanisms of action involve the scavenging of radical oxygen species (ROS), the inhibition of cyclo-oxygenase 1 and 2 and nuclear factor kappa-light-chain-enhancer of activated B cells (NF-кB) pathways, positively antagonizing chronic low-grade inflammation. Among these potential anti-aging compounds, secoiridoid oleuropein (OLE) has largely been studied for its multiple protective effects against aging [11], interfering with the production of inflammatory mediators (anti-inflammatory effect) and directly scavenging free radicals [9, 12].

In this context, our study aimed to investigate: the impact of aging on the T cell immunophenotype of young and older adult individuals; whether influenza vaccination influences the T cell immunophenotype in an age-dependent manner; and the possible anti-inflammatory and antioxidant effects of OLE, both alone and in association with BIRB 796, on T cells in response to influenza virus antigenic stimulation, with the aim of proposing them as vaccine adjuvants.

At first, peripheral blood mononuclear cells (PBMCs), isolated from 52 healthy young (age range 21–35) and older donors (> 60 years old), were analyzed for the expression of principal markers of T cell subpopulations, with particular regard to memory T cell and immunosenescence markers, before (time 0, T0) and after (time 1, T1) immunization with tetravalent influenza vaccine. Antibody titers, referred to as antigens of influenza virus strains included in the vaccine, were also measured in the serum samples obtained from the same recruited cohort. Afterwards, PBMCs, isolated at T0 and T1, were stimulated with specific pools of flu peptides, in combination and without OLE and BIRB 796, to test their pro-, anti-inflammatory and anti-oxidative effects on T cell cytokine secretion.

## Materials and methods

### Cohort description and sampling

A cohort of 52 subjects, 26 young (age range 21–35, 14 females and 12 males) and 26 older (> 60 years old, 13 females and 13 males) were recruited and vaccinated against influenza, with Flucelvax^®^ Tetra, from October to December 2020 at the “Paolo Giaccone”, University Hospital, Palermo. Flucelvax^®^ Tetra contains surface antigens from different inactivated influenza A and B virus strains (A/Hawaii/70/2019 (H1N1)pdm09-equivalent strain (A/Nebraska/14/2019, wild type); A/Hong Kong/45/2019 (H3N2)-equivalent strain (A/Delaware/39/2019, wild type); B/Washington/02/2019-equivalent strain (B/Darwin/7/2019, wild type); B/Phuket/3073/2013-equivalent strain (B/Singapore/INFTT-16-0610/2016, wild type, formulation achieved by database of AIFA - Ricerca Farmaco), chosen based on the official recommendation for the annual flu season. The protocol study was approved by The Ethics Committee of Palermo University Hospital (Improved vaccination Strategies for Older Adults (ISOLDA) - SEP-210574926, No. 01/2020). The study was performed in accordance with the Declaration of Helsinki and its amendments.

Blood samples of each donor were drawn by venipuncture in the morning after a fasting period of 12 h, before (T0), 21–28 (T1) and 56 (time 2, T2) days after influenza vaccination (see Additional Fig. 1 for the recruitment times). The blood was collected in specific tubes containing ethylene diamine tetraacetic acid (EDTA) or no additives. The serum was separated by blood centrifugation of dry tubes and stored at -80 °C. All donors signed informed consent and a detailed anamnestic questionnaire was submitted. To respect privacy, the volunteers were identified by an alphanumeric code. All study participants were healthy donors and their health status was evaluated by common hematochemical analysis. They had no known history of any significant systemic diseases including, but not limited to, immunodepression, kidney or liver diseases, cancer, or autoimmune disorders. Subjects with daily use of immunomodulatory drugs and/or seropositive for Severe acute respiratory syndrome coronavirus 2 (SARS-CoV-2) infection were also excluded. All healthy donors have been tested negative for SARS-CoV-2 IgM and IgG at T0 and T2 to exclude interferences by the novel infection, and for hepatitis B virus (HBV) and human immunodeficiency viruses (HIV) at T0, to exclude the presence of chronic infections with clinically significant immunodeficiency consequences. One young donor was excluded from the analysis because tested positive for SARS-CoV-2 to the serological assay at the follow-up (T2). The characteristics of the aged groups are reported in Table 1. This study is a task of the European Horizon 2020 ISOLDA - Improved vaccination Strategies for Older Adults project- grant agreement No. 848,166.

**Fig. 1:**
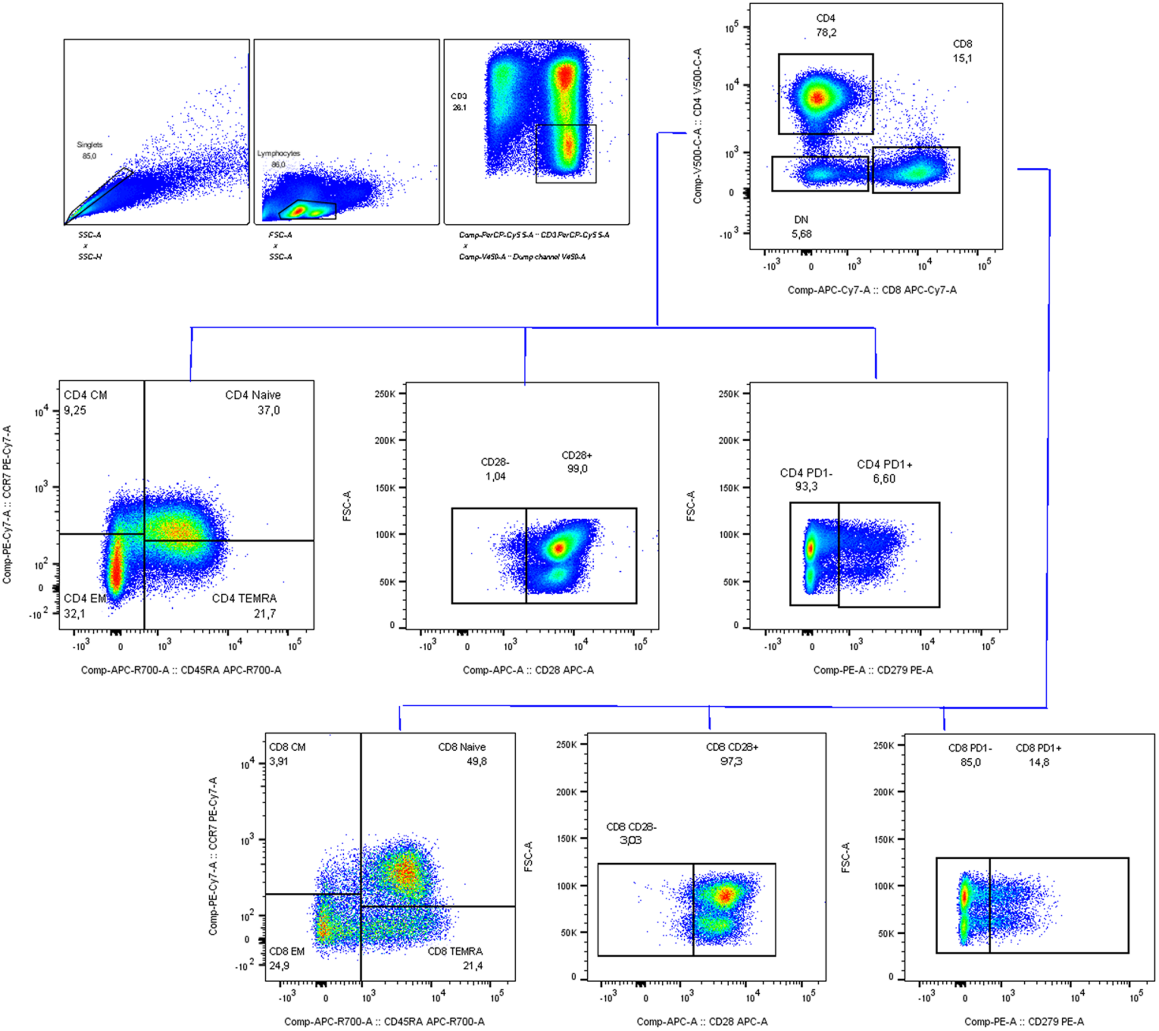
Example of gating strategies used for immunophenotype analysis on PBMCs collected from 20 subjects, including 9 young individuals (aged 18–35 years) and 11 older adults (aged > 60 years), before the administration of the influenza vaccine (T0) and 21–28 days post-vaccination (T1)

**Table 1:** Characteristics of enrolled healthy donors (percentage relative to the numbers are shown in the parentheses in italic)

	Young (*n* = 26)	Older adults (*n* = 26)
**Age (years)**		
Mean ± SD	28 ± 4	68 ± 4
Range	21–35	60–78
**Gender n** (***%***)		
Female	13 (*50*)	13 (*50*)
Male	13 (*50*)	13 (*50*)
Disease n (*%*)		
Cardiovascular (i.e., hypertension, atrial fibrillation)	0 (*0*)	4 (*15*)
Diabetes mellitus	0 (*0*)	2 (*7*)
Hypothyroidism	0 (*0*)	4 (*15*)
Flu-like symptoms post-vaccination n (i.e., mucus, fever, allergic symptoms, mild bronchitis, redness in the site of vaccination) (*%*)	4 (*14*)	7 (*26*)

### Characterization of humoral immune responses of influenza cohort samples

The characterization of the humoral immune response of our cohort was conducted by Bernhard Nocht Institute for Tropical Medicine (member of ISOLDA consortium) on serum samples obtained at T0, T1 and T2. The antibody titer was determined against the following influenza antigens: A/Victoria/2454/2019 (IVR-207) H1N1, A/Hong Kong/2671/2019 (IVR-208) H3N2, B/Phuket/3073/2013 and B/Washington/02/2019. These antigens correspond with those introduced in the vaccine formulation: A/Hawaii/70/2019 (H1N1) pdm09-equivalent strain (A/Nebraska/14/2019, wild type); A/Hong Kong/45/2019 (H3N2)-equivalent strain (A/Delaware/39/2019, wild type); B/Washington/02/2019-equivalent strain (B/Darwin/7/2019, wild type); B/Phuket/3073/2013-equivalent strain (B/Singapore/INFTT-16-0610/2016, wild type, formulation achieved by database of AIFA - Ricerca Farmaco).

### PBMCs isolation

PBMCs were freshly isolated from 50 ml EDTA blood on the day of blood donation by Ficoll-Paque (GE Healthcare, USA) density gradient centrifugation, according to manufacturer instructions. Isolated PBMCs were stained with a 0.4% trypan blue solution (Thermo Fisher Scientific) and then enumerated and assessed for viability using an automated Cell Counter (Invitrogen by Thermo Fisher Scientific). PBMCs were frozen in a freezing medium containing 90% fetal bovine serum and 10% dimethyl sulphoxide and stored in liquid nitrogen before being used for immunophenotypic analyses and cell cultures.

### Multiparametric flow cytometry analysis

Flow cytometry analyses were performed using frozen PBMCs before (immunophenotype characterization of T cells) and after (surface and intracellular cytokine staining) the in vitro stimulation. The acquisition took place at the Central Laboratory of Advanced Diagnosis and Biomedical Research (CLADIBIOR), University Hospital, “P. Giaccone”, Palermo. At least 500.000 events for each sample were acquired on a FACSCanto™ II (BD Biosciences), with a FSC threshold of 10,000 events. The instrument was calibrated using a standard protocol. Voltages were set based on the manufacturer’s recommendations and adjusted using unstained and fluorescence minus one control.

#### Immunophenotype characterization of T cells

Immunophenotype analyses were performed on a cohort of PBMCs selected from the population of recruited subjects. Subject selection for each experiment was performed randomly to ensure balanced and representative sampling, while also considering experimental requirements and sample quality. More specifically, 20 subjects—9 young (18–35 years old) and 11 older (> 60 years old)—were included at both T0 and T1, with inclusion criteria based on cell viability and the number of events detected during flow cytometry analysis. 70% of this selected cohort tested positive for CMV IgG antibody titers. After thawing and washing, PBMCs were surface stained with optimal dilutions of PerCP-Vio700-conjugated anti‐CD3 (clone REA613), VioGreen‐conjugated anti‐CD4 (clone REA623), APC-Vio770‐conjugated anti‐CD8 (clone BW135/80), VioBright R720‐conjugated anti‐CD45RA (clone REA1047), PE-Vio770‐conjugated anti‐CD197 (clone REA546), VioBlue‐conjugated anti‐CD19 (clone REA675), FITC‐conjugated anti‐CD152 (clone REA1003), PE‐conjugated anti‐CD279 (clone REA1165), and APC‐conjugated anti‐CD28 (clone REA612), all from Miltenyi Biotec. Nonviable cells were excluded from the analysis with the Viobility 405/452 Fixable Dye, according to the manufacturer’s instruction (Miltenyi Biotec). An exemplificative schematic representation of the applied gating strategy is shown in Fig. 1. After excluding doublets, lymphocytes were identified based on physical parameters, including forward and side scatter. The CD3 + live population was selected by excluding non-viable cells, which were included in a dump channel alongside CD19 to simultaneously exclude B cells. CD4 + and CD8 + T cell events were subsequently gated on a CD4/CD8 dot plot within the CD3 + live population. Based on the surface marker CD197 (CCR7) and CD45RA, CD4 + and CD8 + T cell populations were divided into CCR7+/CD45RA + naïve, CCR7+/CD45RA- central memory (TCM), CCR7-/CD45RA- effector-memory (TEM) and terminally differentiated CCR7-/CD45RA+ (TEMRA). CD28 and CD279 (Programmed death (PD1)) expressions on CD4 + and CD8 + T cells were also evaluated.

### PBMCs stimulation with PepTivator® influenza a peptide pools for T cell activation and surface and intracellular cytokine staining

**Fig. 2:**
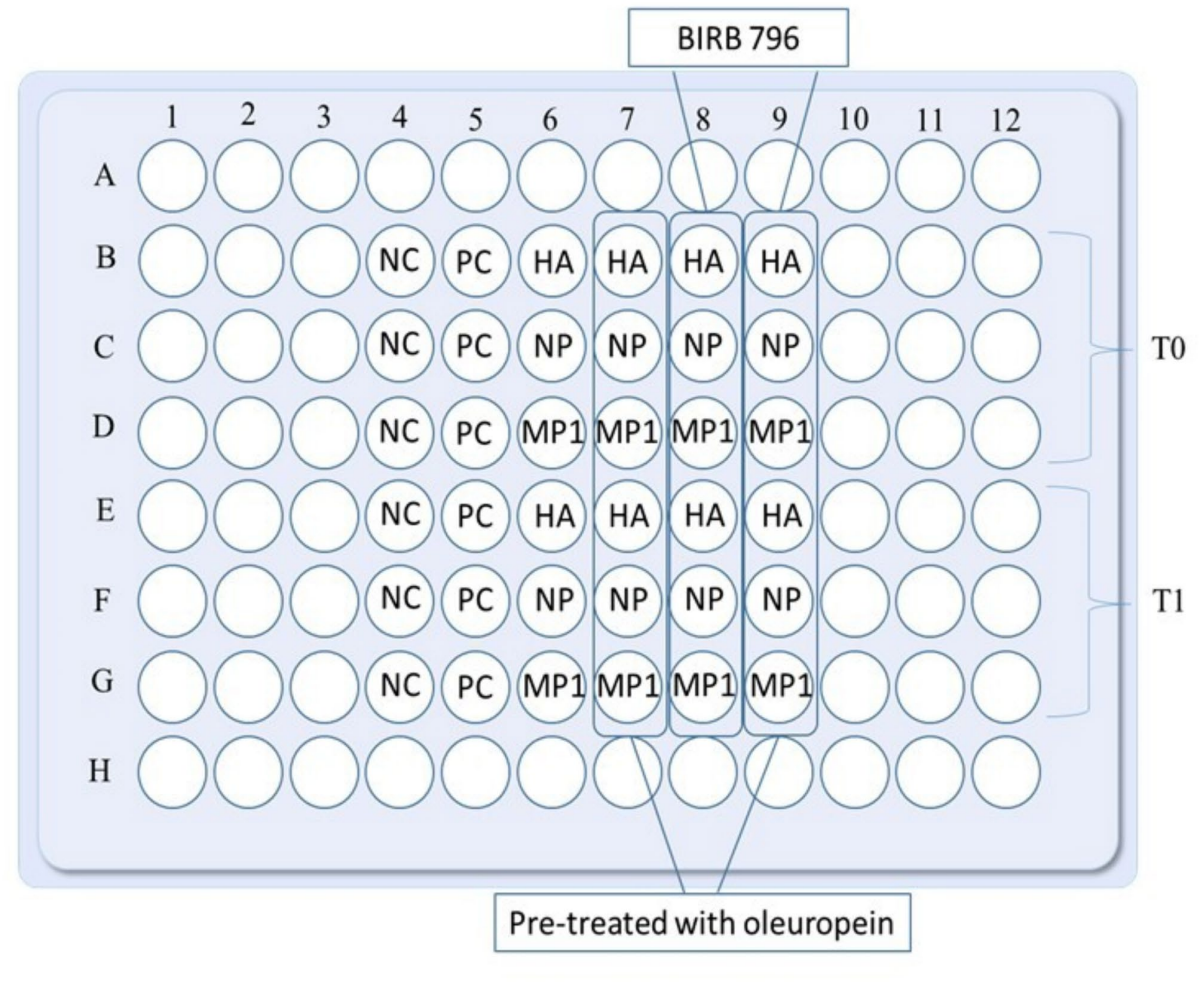
96-well plate template showing culture conditions tested for each donor. HA = hemagglutinin protein pool of peptides; NC = negative control; NP = nucleocapsid protein pool of peptides; MP1 = matrix protein 1 pool of peptides; PC = positive control

**Fig. 3:**
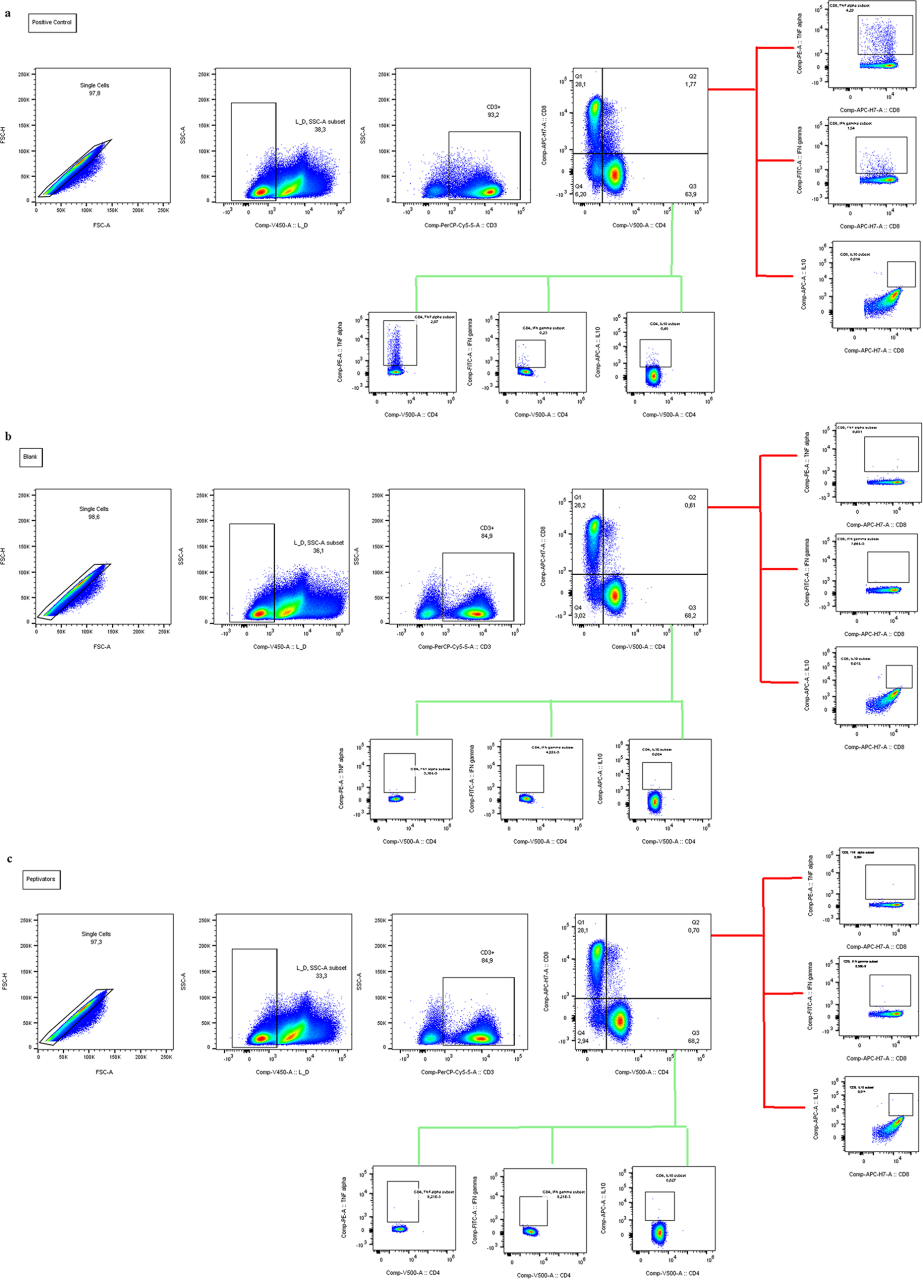
Examples of gating strategies for identifying cytokine-positive CD4 + and CD8 + T cells (TNF-α, IFN-γ, and IL-10) following in vitro stimulation. **a**) Positive control (Cytostim); **b**) Blank (Unstimulated cells); **c**) Peptivators (PepTivator^®^ Influenza A peptide pools)

Based on preliminary test data (see Additional File 1), 15 PBMCs samples were selected from the population of recruited subjects. Subject selection for each experiment was performed randomly to ensure balanced and representative sampling, while also considering experimental requirements and sample quality. In detail, 7 young and 8 older donors were plated at a density of 1 × 10⁶ cells per well in U-bottom 96-well plates at time points T0 and T1. For each donor, PBMCs were thawed, washed, counted with an automatic cell counter, and resuspended in complete Iscove medium (Sigma-Aldrich) containing 5% human serum. After resting for 2 h, the experiments to test the anti-inflammatory and antioxidant role of OLE and BIRB 796 were conducted. A pretreatment with 10 µM OLE was performed by incubating PBMCs for 30 min, at 37 °C in 5% CO₂. This step was conducted to increase the bioavailability of the bioactive compound and priming the cells for better responsiveness to subsequent treatments. Then, the same PBMC were stimulated with 0.6 nmol of three different viral peptides (PepTivator^®^ Influenza A [Miltenyi], which include: hemagglutinin protein (HA), nucleocapsid protein (NP), and matrix protein 1 (MP1), according to manufacturer instructions (see 2.5.2 paragraph). These peptides were tested separately. PBMCs were incubating with them for 2 h, alone and in combination with 800 nM BIRB 796 (Tocris). Unstimulated cells in complete medium served as a negative control for all the conditions. CytoStim™ (Miltenyi Biotec) was used as a positive control. The basal stimulus was given by the cells stimulated only with the three different viral peptides described above (the conditions used for each donor are shown in Fig. 2). After 2 h of stimulation, a protein transport inhibitor cocktail (eBioscience™ Protein Transport Inhibitor Cocktail [500X], Invitrogen) was added, and cells were incubated for an additional 16 h (overnight) at 37 °C in 5% CO₂, as recommended by the manufacturer. Following overnight incubation, cells were harvested and transferred into flow cytometry tubes for surface and intracellular staining. Cells stimulated separately with HA, NP, and MP1 were pooled in single tubes for each treatment condition: one with a peptide mix and OLE, another with the mix and BIRB 796, and a third with the mix and both OLE and BIRB 796. Flow cytometry tubes were also prepared for the control conditions assessed above. We performed extra- and intracellular staining using the following antibodies from Miltenyi Biotec: PerCP-Vio700-conjugated anti‐CD3 (clone REA613), VioGreen‐conjugated anti‐CD4 (clone REA623), APC-Vio770‐conjugated anti‐CD8 (clone BW135/80), PE‐conjugated anti‐Tumor Necrosis Factor (TNF)-α (clone REA656), FITC‐conjugated anti‐Interferon (IFN)-γ (clone REA600), and APC‐conjugated anti‐Interleukin (IL)-10 (clone REA842). Nonviable cells were excluded from the analysis using Viability 405/452 Fixable Dye according to the manufacturer’s instructions. The gating strategy for positive control, unstimulated (blank), and stimulated (PepTivator^®^ Influenza A peptide pools) samples is illustrated in Fig. 3. Briefly, for all conditions, singlets were excluded, followed by the selection of live cells, from which the CD3 + population was identified. CD4 + and CD8 + T cells were subsequently gated from the CD3 + population, and within each subset, cytokine-producing cells were determined. Data from unstimulated samples were subtracted from each treatment condition during the analysis.

#### Oleuropein extracts

OLE used in this study was derived from olive leaves of the *Olea europaea* L., by the Organic Chemistry Laboratory of University Magna Graecia, in Catanzaro. Olive leaves were dried for 48 h at 50 °C, milled, and extracted in an Anton Paar Synthos 3000 MW Oven at 800 W (P-controlled mode) for 10 min in water as a solvent. The leaves were filtered, and the solution was dried under pressure. The mixture was treated with acetone and purified from solid residue by filtration. The solution was evaporated under reduced pressure and the crude product was purified by flash chromatography on silica cartridges (CH2Cl2/MeOH 8:2). OLE was obtained at an High-Performance Liquid Chromatography purity of 98%. Analytical data of the pure OLE were compared with data reported in the literature.

#### Influenza a peptide pools

Peptides used in this study for the in vitro stimulation of antigen-specific CD4 + and CD8 + T cells consisted of 3 different pools of lyophilized peptides, as 15-mer sequences with an 11 amino acid overlap, covering respectively the sequence of the HA (PepTivator^®^ Influenza A HA, Miltenyi Biotec), NP (PepTivator^®^ Influenza A NP, Miltenyi Biotec) and of the MP1 (PepTivator^®^ Influenza A MP1, Miltenyi Biotec) of the human Influenza A virus (H1N1) (average purity > 70%). The lyophilized peptides were reconstituted with sterile water, following the manufacturer’s guidelines.

The peptide pool utilized is specifically designed to analyze the in vitro efficacy of stimulation of antigen-specific CD4 + and CD8 + T cells. In this study, the PepTivator^®^ peptides are derived from antigens of the H1N1 influenza virus, a strain included in the vaccine formulation used for participant recruitment. Notably, during the 2019–2020 influenza season, influenza A(H1N1)pdm09 viruses were the most commonly reported influenza viruses, making this strain particularly relevant for this study.

### The ROS and RNS test

The OxiSelectTM in vitro ROS/RNS assay kit (Cell Biolabs, Inc.) has been used for detecting hydrogen peroxide, peroxyl radicals, nitric oxide, and peroxynitrite anions on cell supernatants after T cell stimulation with the pool of viral peptides and for evaluating antioxidant’s effect of OLE and BIRB 796 on these free radical molecules. Briefly, after an incubation of the samples with a catalyst, the dichlorodihydrofluorescein probe was added to all wells, promoting the oxidative process. Fluorescence intensity, proportional to the total ROS/RNS levels within the sample, was measured in a 96-well plate and detected with Synergy HT (software version 2.01.14) at 485/20–528/20 nm. Data are shown in Relative Fluorescence Units (RFU).

### Statistical analysis

Flow cytometry data were analyzed using FlowJo version 10.5.3 (Tree Star, Inc., Ashland, OR, USA). Immunophenotype, comparison of cytokine production and ROS/RNS levels between age groups at the two recruitment times (T0 e T1) was performed by a One and 2-way ANOVA test for multiple comparisons (GraphPad Prism version 9.3.1 - GraphPad Software, San Diego, CA, USA). The Bonferroni correction was applied to account for the multiple comparisons performed in the study. This adjustment is necessary to mitigate the increased likelihood of false positives that arise when multiple statistical tests are conducted simultaneously. The use of multiple comparison techniques enables us to perform a single comprehensive test to evaluate the effect of recruitment timing across the different treatment strategies employed in the in vitro analysis.

## Results

### Characterization of humoral immune responses of influenza cohort samples

**Fig. 4:**
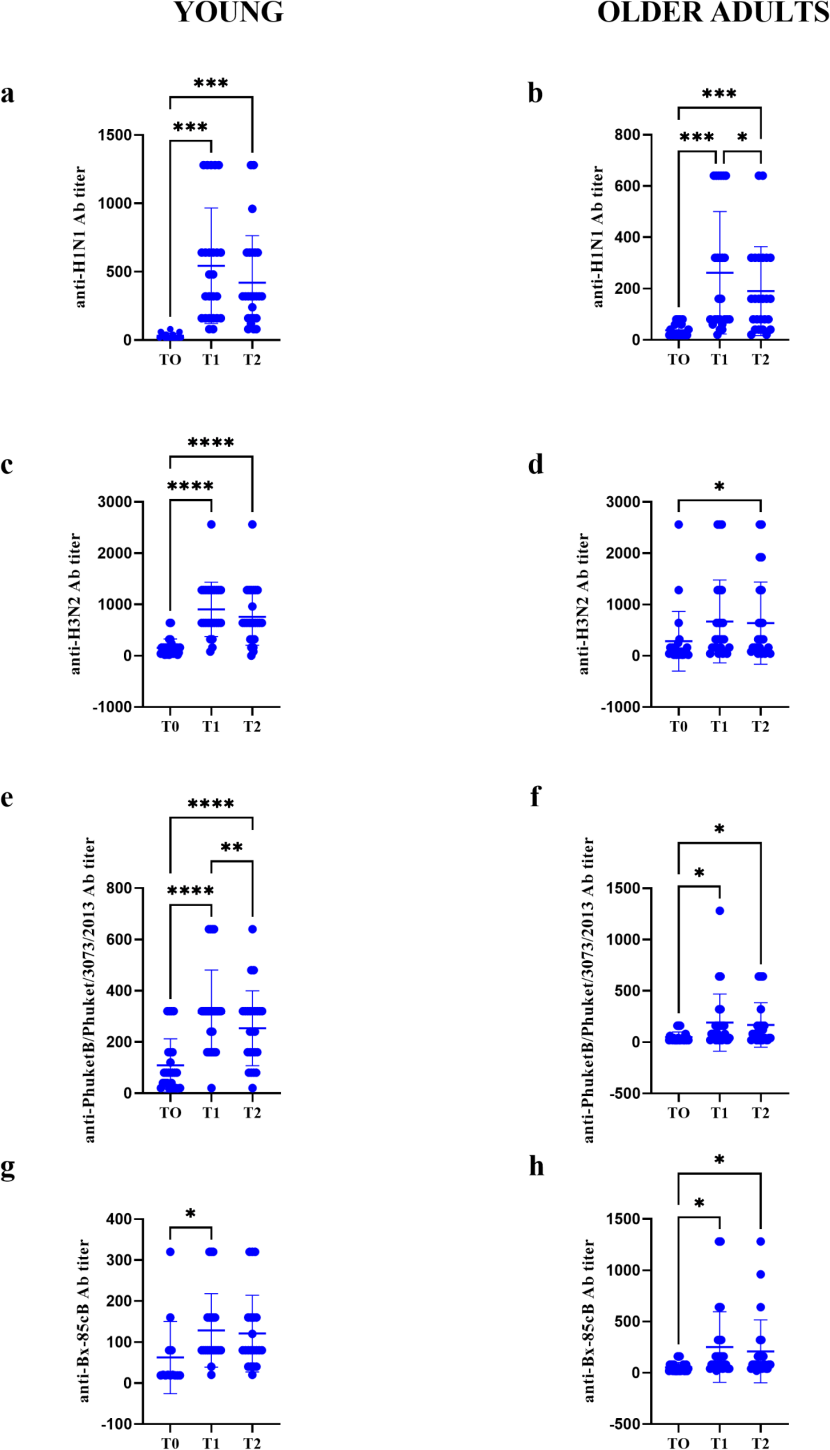
Description of antibody titers at different recruitment time points across age groups. Age group of young: **a**, anti-H1N1 Ab titer; **c**, anti-H3N2 Ab titer; **e**, anti-PhuketB Ab titer; **g**, anti-Bx-85cB Ab titer. Age group of older adults: **b**, anti-H1N1 Ab titer; **d**, anti-H3N2 Ab titer; **f**, anti-PhuketB Ab titer; **h**, anti-Bx-85cB Ab titer. Ab = antibody; T0 = Time zero; T1 = Time 1 (21–28 days after vaccination); T2 = Time 2 (56 days after vaccination)

Antibody titers against the influenza antigens A/Victoria/2454/2019 (IVR-207) H1N1, A/Hong Kong/2671/2019 (IVR-208) H3N2, B/Phuket/3073/2013 and B/Washington/02/2019, showed a significant increase in antibody levels at T1, and a subsequent decrease at T2, compared to T0, except in the young group for anti-B/Phuket/3073/2013, which showed a significant increase at T2, and for anti-Bx-85cB, where the significance at T2 was absent (Fig. 4e-g). In the older adults group, all tested strains showed results similar to those observed in the young group, except for anti-A/Hong Kong/2671/2019 (IVR-208) H3N2, which exhibited a significant increase in antibody titer at T2 (Fig. 4d). Focusing on the viral strain H1N1, for which the in vitro T cell response is elicited, significant differences were found in both young and older adult groups between T0 and T1-T2, and between T1 and T2 respectively (Fig. 4a and b; young individuals: p-value T0 vs. T1 = 0.0001, p-value T0 vs. T2 = 0.0002; older individuals: p-value T0 vs. T1 = 0.0003, p-value T0 vs. T2 = 0.0007, p-value T1 vs. T2 = 0.025).

**Fig. 5:**
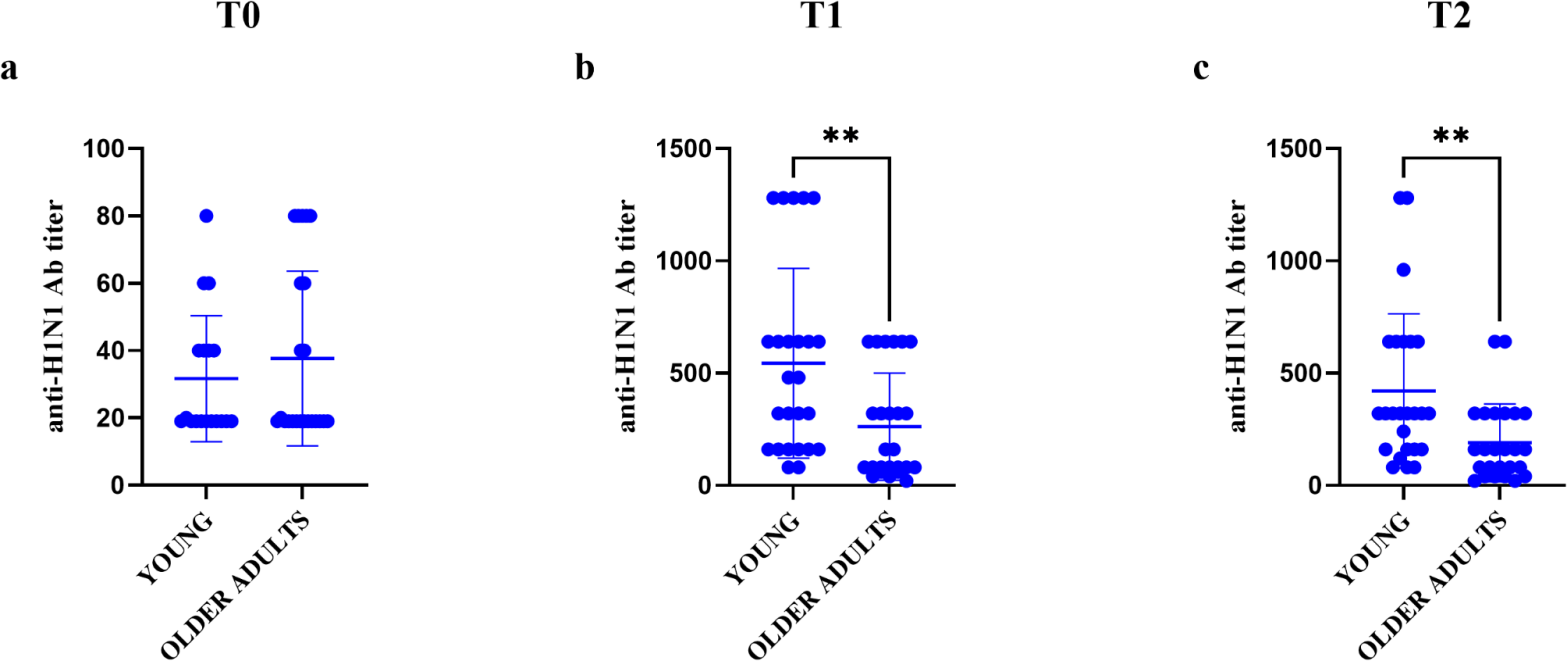
Comparison of H1N1 antibody titers across age groups based on different recruitment times. **a**, T0; **b**, T1; **c**, T2. Ab = antibody; T0 = Time zero; T1 = Time 1 (21–28 days after vaccination); T2 = Time 2 (56 days after vaccination)

Comparing the two reference populations of young and older individuals for anti-H1N1 titers, at T0 the antibody titer was higher in older individuals, although not statistically significant, suggesting a higher basal response to this viral antigen (Fig. 5a). Statistically significant differences were observed only at T1 and T2 (Fig. 5b and c; p-value T1 = 0.0055, p-value T2 = 0.0043), with the titer of the older group being lower than that of the younger one.

### Immunophenotype characterisation of T cells

**Fig. 6:**
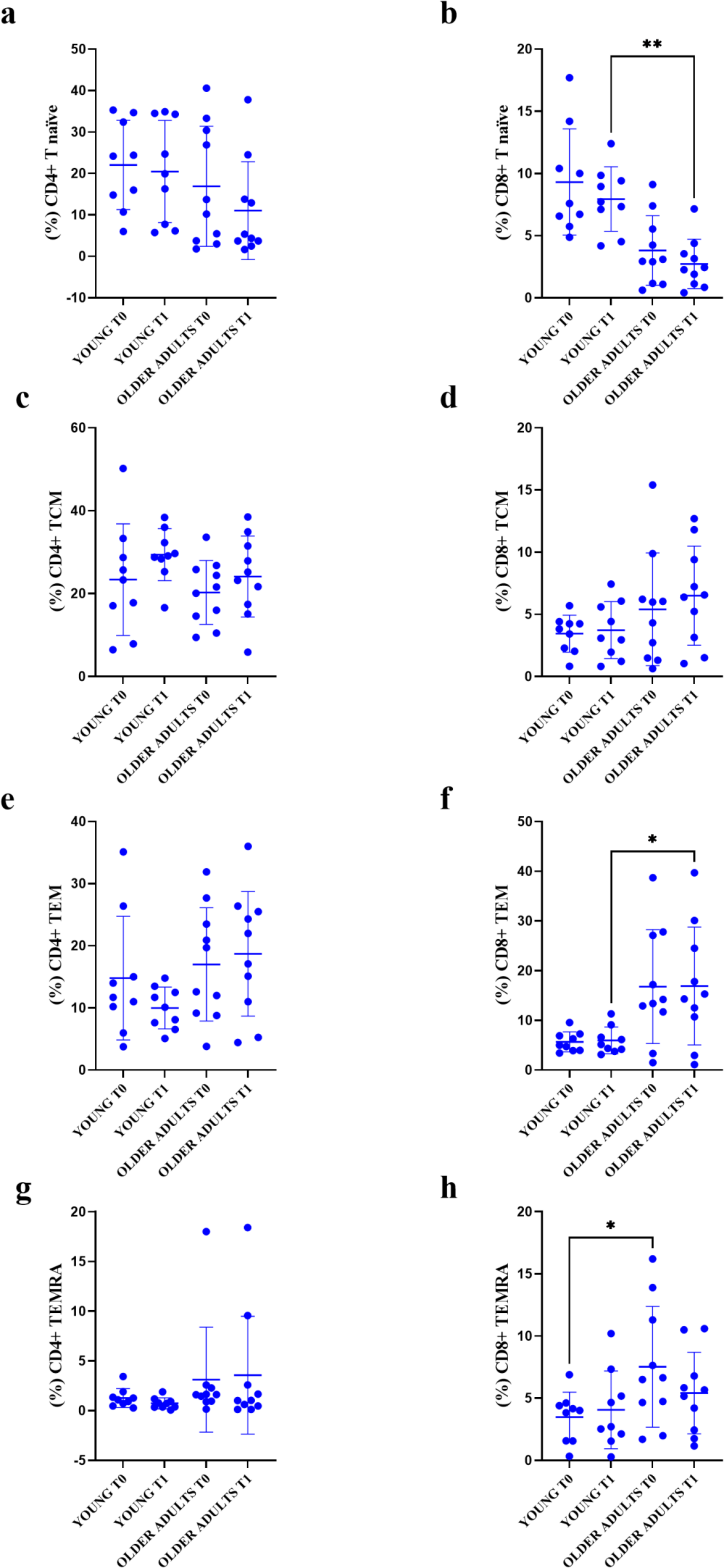
Comparison of the young and older adults age groups and recruitment time points (T0 and T1) within CD4 + and CD8 + T cell populations. **a**, CD4 + naïve; **c**, CD4 + TCM; **e**, CD4 + TEM; **g**, CD4 + TEMRA; **b**, CD8 + naïve; **d**, CD8 + TCM; **f**, CD8 + TEM; **h**, CD8 + TEMRA; TCM = T central memory; TEM = T effector memory; TEMRA = T effector memory cells re-expressing CD45RA; T0 = Time zero; T1 = Time 1 (21–28 days after vaccination)

At T0 and T1, the analysis of T cell subsets from young and older individuals confirmed an age-related decrease trend in CD4 + and CD8 + naïve T cells (Fig. 6a and b). Analyzing the cell percentages, we found a slight reduction in CD4 + and CD8 + naïve T cells at T1, compared to T0, between young and older adults groups, with a statistical significance observed only for the CD8 + naïve T cells and for T1 time of recruitment (p-value T1 young vs. T1 old = 0.004) (Fig. 6b).

A not statistically significant increase was observed for CD4 + and CD8 + TCM cells between T0 and T1 (Fig. 6c). For CD8 + TCM higher percentages for the older adults could be observed without statistical relevance (Fig. 6d).

The percentage of CD8 + TEM was significantly higher for older adults at T1 (p-value T1 young vs. T1 old = 0.039) (Fig. 6f).

Finally, there was observed a decrease in the percentage of CD8 + TEMRA in old people at T1 vs. T0, with a statistically significant increase only between young and older adults at T0 (p-value = 0.0438) (Fig. 6h). While CD8 + TEMRA cells decrease between T0 and T1 in older adults, the reduction is not statistically significant (Fig. 6h). In contrast, a slight increase is observed in younger individuals (Fig. 6h). Nonetheless, the levels in older adults remain higher than those in younger individuals, even for CD4 + TEMRA cells (Fig. 6g).

Regarding the markers of exhaustion (PD-1), there is no significant increase in its frequency in old subjects at T1 within the CD8 + T cell population. For CD28, chosen as senescent-related marker, no significant differences were observed (data not shown).

### PBMCs stimulation with PepTivator® influenza a peptide pools for T cell activation

**Fig. 7:**
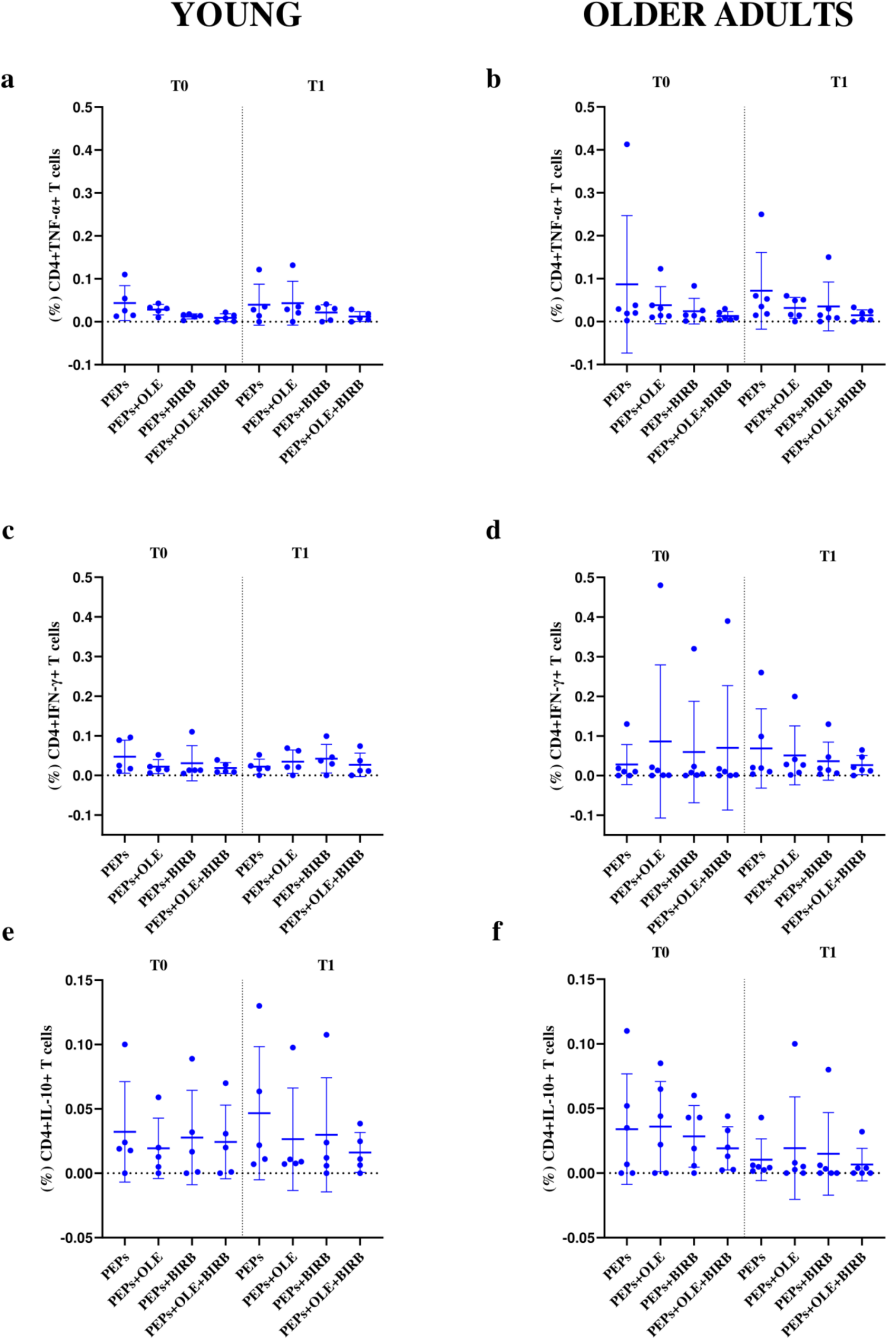
Description of cytokine-producing CD4 + T cell populations in the different culture conditions. Young age group: **a**, CD4 + TNF-α + T cells; **c**, CD4 + IFN-γ + T cells; **e**, CD4 + IL-10 + T cells. Older adults age group: **b**, CD4 + TNF-α + T cells; **d**, CD4 + IFN-γ + T cells; **f**, CD4 + IL-10 + T cells. PEPs = PepTivator^®^ Influenza A (basal stimulus); OLE = oleuropein; BIRB = BIRB 796; OLE + BIRB = combination of oleuropein + BIRB 796; PEPs + OLE/BIRB/OLE + BIRB = PepTivator^®^ Influenza A + oleuropein/BIRB 796/oleuropein + BIRB 796

**Fig. 8:**
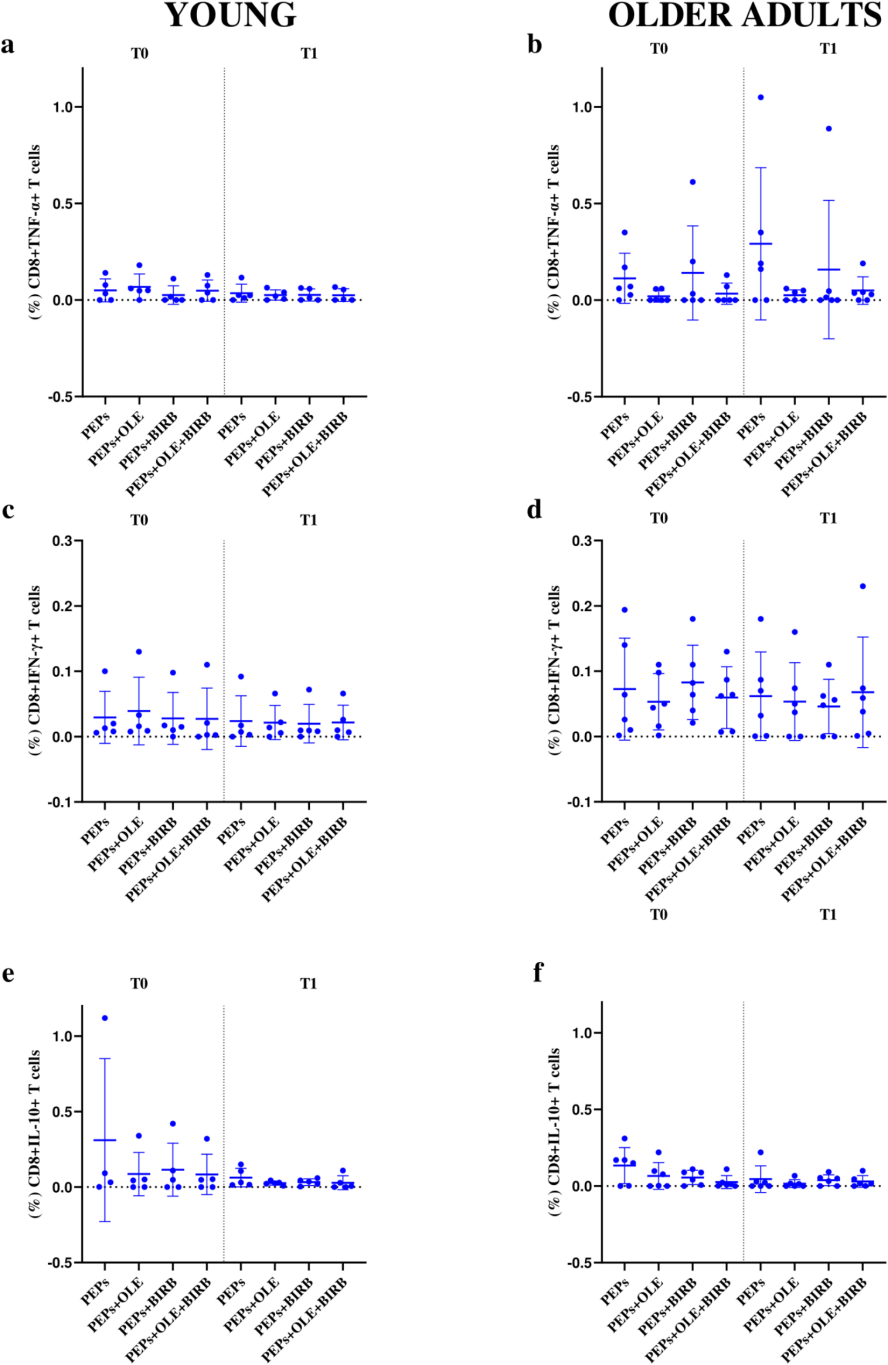
Description of cytokines producing CD8 + T cell populations in culture conditions. Young age group: **a**, CD8 + TNF-α + T cells; **c**, CD8 + IFN-γ + T cells; **e**, CD8 + IL-10 + T cells. Older adults age group: **b**, CD8 + TNF-α + T cells; **d**, CD8 + IFN-γ + T cells; **f**, CD8 + IL-10 + T cells. PEPs = PepTivator^®^ Influenza A; OLE = oleuropein; BIRB = BIRB 796; OLE + BIRB = combination of oleuropein + BIRB 796; PEPs + OLE/BIRB/OLE + BIRB = PepTivator^®^ Influenza A + oleuropein/BIRB 796/oleuropein + BIRB 796

Based on the analysis of the percentage of CD8+/CD4 + IL-10+/IFN-γ+/TNF-α + T cell populations, no statistically significant differences were observed between T0 and T1 for any of the tested conditions, including treatment with OLE and/or BIRB 796 compared to the basal stimulus condition represented by PepTivator^®^ Influenza A peptide pools (PEPs), within each group. However, in the young and older adults groups, at T0, a greater reduction of CD4 + TNF-α + T cell levels was shown in presence of OLE + BIRB 796 and BIRB 796 (Fig. 7a and b). Whereas, at T1, in both age groups, the reduction seemed more evident only in the presence of OLE + BIRB 796 (Fig. 7a and b). For CD8 + TNF-α + T cells, higher values could be observed in the older adults group compared to the young ones for every condition and timing tested (Fig. 8a and b). A potential effect of OLE alone or in combination with BIRB 796 on the reduction of CD8 + TNF-α + T cells was noted at both T0 and T1 only for the older adults group (Fig. 8b). No conclusions could be drawn about the effects of the different evaluated stimuli in the young group, in which OLE seemed to stimulate this cell population and there was not a very different trend between the mentioned conditions (Fig. 8a).

In young samples, at T0, a greater reduction in CD4 + IFN-γ + T cell values was observed compared to the baseline stimulation, in the presence of OLE alone and OLE + BIRB 796 treatments (Fig. 7c). However, this reduction did not occur at T1. In the older adults group, at T0, OLE seems to induce an increase in the percentage of CD4 + IFN-γ + T cells concerning the baseline, whereas at T1, compared to the baseline condition, the OLE + BIRB 796 treatment resulted in a reduction in the CD4 + IFN-γ + T cell population than either BIRB 796 or OLE alone, indicating a synergistic effect of the combined stimulation (Fig. 7d). For the CD8 + IFN-γ + T cells population no relevant conclusions could be derived for the young population group compared with the baseline stimulus (Fig. 8c). In older adults, OLE appeared to cause a reduction in CD8 + IFN-γ T cells at both T0 and T1 in a not statistically significant manner, while BIRB 796 showed an opposite effect between T0 and T1 (Fig. 8d).

Ultimately, OLE likely induced a non-statistically significant increase in the percentage of CD4 + IL-10 + T cells at both T0 and T1, compared to the PEPs stimulation condition and other treatments, including BIRB 796, alone or in combination with OLE, in the older adults group (Fig. 7f). In the younger group, OLE, alone or in combination, appeared to reduce CD4 + IL-10 + T cells at both T0 and T1 (Fig. 7e). For CD8 + IL-10 + T cells, no clear increase in this population was observed in either the young or older adult groups, which would suggest a possible anti-inflammatory effect related to this cytokine. However, it seemed that OLE, alone or in combination with BIRB 796, was associated with a reduction of this population at T0, with the response converging at T1 (Fig. 8f).

### ROS and RNS analysis

**Fig. 9:**
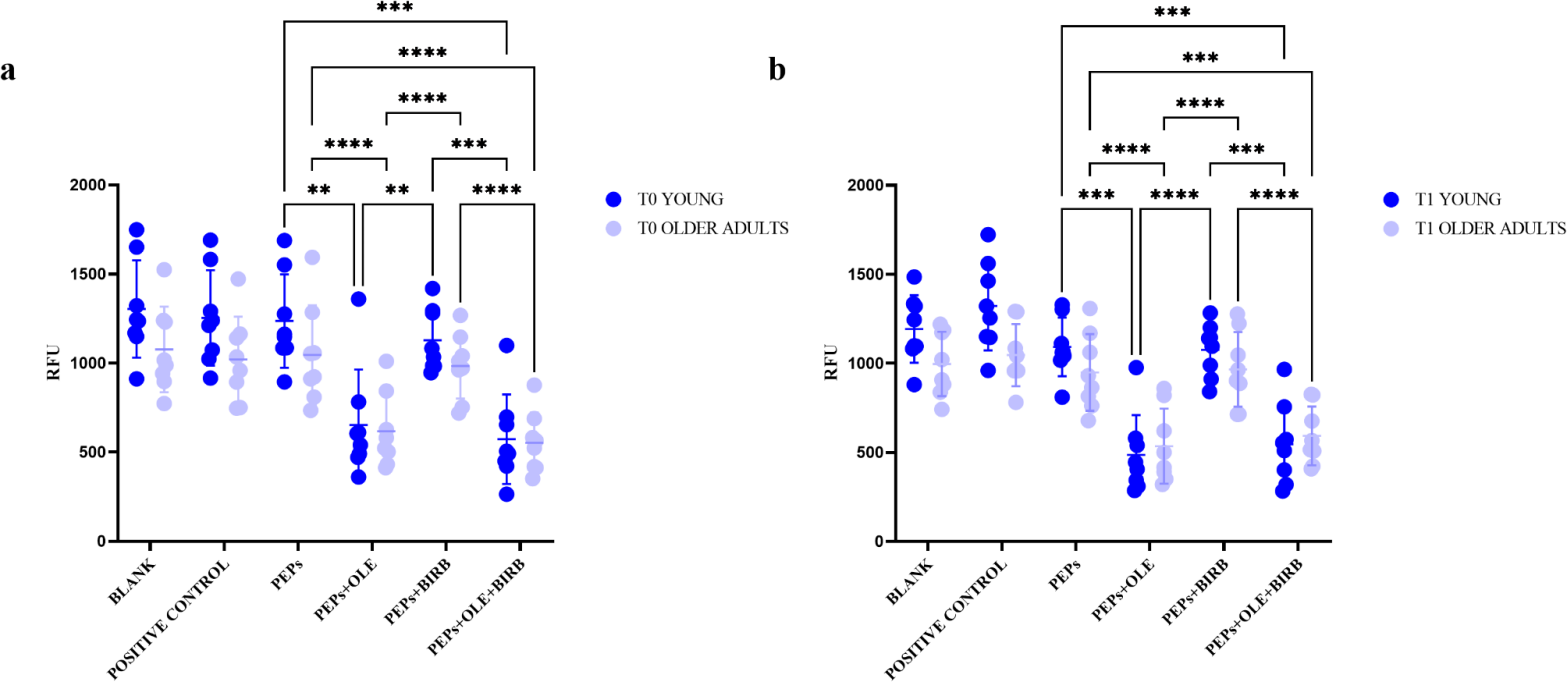
Comparison of RFU levels of ROS/RNS across different culture conditions in the two age groups. **a**, T0; **b**, T1. RFU = relative fluorescence units. PEPs = PepTivator^®^ Influenza A; OLE = oleuropein; BIRB = BIRB 796; OLE + BIRB = combination of oleuropein + BIRB 796; T0 = Time zero; T1 = Time 1 (21–28 days after vaccination)

The analysis of RFU levels showed T0 lower levels of ROS/RNS compounds in older adults than in young individuals for all tested conditions (Fig. 9a and b). Focusing on OLE treatment, in both the young and the older adult groups, a significant reduction in RFU is observed at T0 (Fig. 9a) and T1 (Fig. 9b) with OLE alone or in combination with BIRB 796 (T0, young group: p-value PEPs vs. OLE treatment = 0.019; p-value PEPs vs. OLE + BIRB 796 treatment = 0.0001; T0, older adults group: p-value PEPs vs. OLE treatment < 0.0001; p-value PEPs vs. OLE + BIRB 796 treatment < 0.0001; T1, young group: p-value PEPs vs. OLE treatment = 0.0001; p-value PEPs vs. OLE + BIRB 796 treatment = 0.0004; T1, older adults group: p-value PEPs vs. OLE treatment < 0.0001; p-value PEPs vs. OLE + BIRB 796 treatment = 0.0002). No statistically significant difference can be observed from the comparison between T0 and T1 recruitment in each age group (data not shown).

A summary of the key findings from the study is presented in Table 2.

**Table 2:** The key findings from the analysis conducted

Category	Key results
Antibody Titers	Significant increase in antibody levels at T1, followed by a decrease at T2, except for anti-Bx-85cB in the young group (no significance at T2). Older adults showed higher baseline titers (not significant) but lower titers than young adults at T1 and T2 (*p*-values: T1 = 0.0055, T2 = 0.0043).
T Cell Immunophenotype	Age-related decrease in CD4 + and CD8 + naïve T cells. CD8 + naïve T cells showed significant reduction at T1 in older vs. young adults (*p* = 0.004). CD8 + TEM were higher in older adults at T1 (*p* = 0.039). CD8 + TEMRA decreased in older adults at T1, with a significant T0 difference between young and old (*p* = 0.0438).
PBMCs stimulation with PepTivator^®^ Influenza	OLE and OLE + BIRB 796 reduced CD4+/CD8 + TNF-α + T cells in older adults, with variable trends in young individuals. CD8 + IFN-γ + T cells were higher in older adults across conditions. OLE + BIRB 796 synergistically reduced CD4 + IFN-γ + T cells in older adults at T1. IL-10 trends varied by age and condition, without statistical significance and a clear trend.
ROS/RNS Analysis	Older adults showed lower ROS/RNS levels compared to young individuals across all conditions. OLE and OLE + BIRB 796 significantly reduced ROS/RNS levels in both age groups at T0 and T1 (e.g., T0 older adults: PEPs vs. OLE, *p* < 0.0001). No significant differences were found between T0 and T1 within groups.

## Discussion

Vaccines are the goals of preventive medicine. So, vaccinating vulnerable populations to mitigate the harmful effects of viral diseases, such as influenza, has grown over the years. Despite these efforts, flu-vaccines are less efficient in the older adults, with effectiveness rates of 30–50% for individuals over 65 years old, compared to 70–90% for younger individuals [13]. The observed inefficacy of vaccination in this population is often attributed to the exclusion of frail subjects from clinical trials. This results in a lack of crucial data, especially regarding optimal dosing and the need for booster doses to enhance vaccine coverage and ensure effectiveness. Another key consideration for preventive vaccination is assessing the history of previous infections and vaccinations in older adults patients [14].

Regarding the causes, this mild vaccine response could be linked with the changes that the immune system undergoes during aging, including immunosenescence and inflamm-aging [15, 16]. Immunosenescence leads to significant shifts in the phenotypes and functionality of immune cells, with a reported decrease in naïve T cells and an increase in TEM and TEMRA cells in the old people [8]. Concurrently, inflamm-aging negatively impacts immunity by impairing the capacity of immune cells to respond to new antigen challenges, including vaccination ones [15].

The ISOLDA consortium was established and funded by the European Commission in 2020 to develop new strategies to address the poor efficiency of flu (and other) vaccines. This involves creating formulations with compounds that can mitigate the effects of the exacerbating inflamm-aging after vaccination. More specifically, at first, analyzing the immunophenotype of key subset cells involved in the vaccine response, we aimed to assess how the immune system, particularly T cell subpopulations, changes with age and in response to flu-vaccination. In this regard, some studies have reported a predominance of CD4 + naïve T cells in the immune response to vaccines, while memory CD8 + T cells are more prevalent in long-term defence [17]. Nonetheless, the literature on the effects of influenza vaccination on T cell populations is limited. At a second stage, we tested the effect of OLE and BIRB 796 as possible anti-inflammatory and antioxidant compounds able to reduce the inflammatory and oxidative stress induced by a viral stimulus in cultured T cells, reproducing the oxi-inflammatory stimulus generated by the administration of the vaccine. The combination of these two compounds has never been tested before, and their clinical application, particularly in the context of immune-related therapies such as vaccination, has not yet been developed.

Regarding the immunophenotype analysis, our results demonstrated statistically significant differences in CD8 + naïve T cells, TEM, and TEMRA percentages between the two analyzed age groups (Fig. 6b, f, and h). These findings are consistent with existing literature, describing the shifting distribution of these T cell subsets during the aging process [8]. The reduction in CD8 + naïve T cells with aging could impair the ability of the immune system in older individuals to mount an effective response after vaccination or any antigenic stimulation [18]. Specifically, comparing young to older adult groups, we have shown that CD8 + naïve T cells were significantly reduced, after vaccination, in the older adult group (Fig. 6b). TEM levels were higher at both T0 and T1 in the older population, with a statistically significant difference at T1 compared to the younger group (Fig. 6f). This suggests that the vaccine may play a role in shaping the immune response post-vaccination in older individuals, although our analysis does not confirm a direct change attributable to the vaccination itself because the same trend was observed at T0 although without statistical significance. Furthermore, no significant differences, within the same age group, at the different recruitment times (T0 and T1) were observed (Fig. 6). A distinct trend between T0 and T1 emerges exclusively for CD8 + TEMRA cells (Fig. 6h). In the younger group, these cells increase, whereas the old population exhibit a decrease over the same period (T0 vs. T1), although the difference is not statistically significant. Notably, TEMRA values consistently remain higher in the older adults compared to the young, reaffirming their association with age while suggesting an intriguing hypothesis about the potential effects of vaccination on this specific cell population. The increase in TEMRA cells with aging is closely associated with immunosenescence. However, the observed reduction of TEMRA cells after vaccination could suggest either a reactivation of the immune system or a reprogramming of the immune response toward an antigen-specific focus. Although not statistically significant, a rise in TCM and TEM cells, along with a decrease in naïve cells, was noted between T0 and T1, which appears to contradict the latter hypothesis. Additional experiments investigating the effects of vaccination on the distribution of memory T cells in the old population are needed to better clarify and define this pattern.

Also the analysis of the expression of exhaustion and senescence-like markers (i.e., CD28, and PD-1) could indicate a strongly impaired immune response to vaccination by inhibiting T cell activation and proliferation [13]. However, we did not obtain significant results for these cited markers, leading us unable to verify the contribution of specific immunosenescence factors in the vaccination response [18].

In older individuals, also the response to the vaccine and the duration of long-term protection are affected by the decline in antibody production [18]. In our study cohorts, the antibody response to all strains tested and included in the administered vaccine (Flucelvax^®^ Tetra) remained higher than pre-vaccination levels at later time points for both analyzed populations (Figs. 4 and 5). Notably, in older adults, before vaccination, we observed a higher level of antibody titer against the H1N1 strain compared to younger individuals, although this difference was not statistically significant (Fig. 5a). However, at T1 and T2, respectively 21/28 and 56 days post-vaccination, in the older population there was a statistically significant decline in antibody levels compared to the younger group (Fig. 5b and c) although, within the older adults population group, there is an increase in antibody titer at these times compared to T0 (Fig. 4b) [13]. These data appear to align with findings in the literature about the efficacy of the tetravalent vaccine in eliciting humoral responses despite the age.

It should be emphasized that generally the antibodies titer towards virus strains observed in the older population (aged ≥ 65 years) was lower or comparable with seroconversion rates of younger groups, highlighting the dependence from age of vaccine’s effectiveness [19, 20]. Furthermore, the absence of a clear association between the cellular changes analyzed post-vaccination and antibody titer aligns with other studies that show that antibody titer does not correlate with the cellular response to the virus or vaccination [17, 21]. So, our data should confirm the lower response of older adults compared to the younger population in terms of antibody titers and consequently the probable lower vaccine efficacy linked to the humoral response [22].

Among the proposed strategies to enhance vaccination efficacy in the older adults, one of these is the mitigation of the inflammatory process, partly due to inflamm-aging, partly exacerbated by vaccination, targeting specific inflammation markers [23]. In this context, phenolic compounds from olive oil have demonstrated excellent anti-inflammatory and antioxidant properties in both in vitro and in vivo studies [9, 12]. The use of adjuvants derived from natural sources has become a key area of research, with particular focus on plant-based adjuvants such as saponins, tomatine, and others [24, 25]. These compounds show significant promise due to their dual role as immunopotentiators and anti-inflammatory. In addition, the use of plant-derived adjuvants offers enhanced safety profiles and potential environmental and economic benefits. Specifically, plant-derived adjuvants are highly valued for their biocompatibility, renewability, and ability to reduce reliance on synthetic chemicals, making them a crucial component in promoting sustainable practices [24, 25]. Specifically, in this work, it is hypothesized that OLE acts on the NF-κB pathway by modulating the production of pro-inflammatory (i.e., TNF-α, IL-6, and IFN-γ) and anti-inflammatory (i.e., IL-10) cytokines, as well as factors involved in the oxidative stress response [26, 27]. In particular, OLE has been shown to inhibit NF-κB activation by preventing the phosphorylation and degradation of its inhibitor, IκBα, thereby suppressing the nuclear translocation of NF-κB [11]. Additionally, its antioxidant effects may be linked to the interplay between NF-κB and other pathways, including those involving COX enzymes and Nrf2 [11]. By modulating Nrf2, oleuropein enhances the expression of antioxidant response elements, contributing to its protective effects against oxidative damage and inflammation [11]. Similarly, BIRB 796 has shown effectiveness as an inhibitor of p38 MAPKs, which are molecular targets for reducing inflamm-aging in adults [23, 27–29].

To study the effects on CD8+/CD4 + T cell populations producing key cytokines involved in the inflammatory process (TNF-α, IFN-γ, and IL-10), we tested OLE and BIRB 796, both individually and in combination, on the cultured PBMCs of voluntary donors at two recruitment times (T0 and T1), after the stimulation with a viral peptides stimulus from the H1N1 viral strain (PepTivator^®^ Influenza A). Our investigations gave some explorative results about a possible role of OLE, alone or in combination with BIRB 796, in modulating CD8+/CD4 + TNF-α+/IFN-γ+/IL-10 + T cell levels, particularly in the older adults population, although without any statistical significance evidence (Figs. 7 and 8). The findings on the reduction of TNF-α + T cells by OLE, alone or in combination with BIRB 796, could be interesting due to the role of CD8 + TNF-α + T cells in inducing lung damage during flu infection and contributing to the increased production of pro-inflammatory cytokines and chemokines necessary for immune cell recruitment [17, 30]. Interestingly, the combination of OLE and BIRB 796 resulted in a probably more pronounced reduction in TNF-α levels than either BIRB 796 or OLE alone, suggesting a synergistic effect, attributed to BIRB and OLE’s inhibition of the p38 MAPK and NF-κB pathway respectively, which plays a key role in regulating inflammatory responses. This synergy could likely arise from the complementary mechanisms by which BIRB 796 and OLE impact molecular pathways involved in inflammation and oxidative stress, leading to a more effective suppression of TNF-α.

In the same way, IFN-γ is crucial in the antiviral and vaccination response. Its levels tend to decrease following vaccine administration and correlate with the antibody response in the general population [31]. However, in the older population, IFN-γ can have a negative role due to its ability to amplify the inflammatory process, which underlies the reduced vaccine response. In our analysis, OLE appears to probably act on IFN-γ-producing T cells, particularly in CD8 + T cells in the older adults group at T1 (Fig. 8d). OLE showed stable or slightly increased IFN-γ expression, reflecting OLE’s possible ability to support immune signalling pathways without excessive suppression of IFN-γ but behaving as a hormetic agent (Figs. 7d and 8c). This effect could be beneficial in the induction of virus elimination response during infections or vaccination itself by enhancing the immune response against viral antigens [32]. BIRB 796 did not seem to suppress IFN-γ, suggesting that it should not interfere with immune responses mediated by IFN-γ. However, when the compounds were combined, IFN-γ levels appeared to be maintained or modestly enhanced, which indicates a balanced immune response where inflammation is reduced, without compromising the antiviral and immune regulatory functions of IFN-γ.

Regarding IL-10 modulation, OLE displayed supposed minimal anti-inflammatory effects, as no significant increase in IL-10 + T cells was observed. In the older group, a slight reduction in CD8 + IL-10 + T cells was noted, particularly with OLE alone, suggesting a limited role in countering inflammation through IL-10 pathways (Fig. 8f). Interestingly, in the young group, OLE, in combination with BIRB 796, resulted in a decrease in CD4 + IL-10 + T cells (Fig. 7e), which may favor a pro-inflammatory immune profile more suitable for vaccine responses in this demographic area.

Also oxidative stress regulation involves both the NF-κB and p38 MAPK pathways, which can be activated by ROS/RNS, thereby promoting inflamm-aging. We have hypothesized that OLE and BIRB 796 could have reduced the effects of ROS and directly modulated their production [33–35]. Our analysis indicates that OLE, alone or in combination with BIRB 796, reduces ROS/RNS RFU values in both young and older adult populations (Fig. 9). However, we did not observe any effects on oxidative stress modulation related to the timing of recruitment. This suggests that the combined antioxidant effects of both agents may counteract oxidative stress, but we cannot make conclusions about their antioxidant effects concerning vaccination.

In summary, our exploratory findings suggest a role of OLE and BIRB 796 in the modulation of inflammation and immune responses, which is highly relevant for improved vaccine formulations. OLE could exhibit antioxidant and anti-inflammatory properties while preserving or slightly enhancing IFN-γ levels, a cytokine critical for antiviral immunity and effective vaccine responses. This suggests that OLE may act as a hormetic agent, reducing excessive inflammation while supporting immune signalling pathways that are essential for adaptive immunity. On the other hand, BIRB 796 should demonstrate an anti-inflammatory effect by targeting the p38 MAPK pathway, which is central to regulating inflammatory cytokines such as TNF-α. When combined, OLE and BIRB 796 could exert a synergistic effect, significantly reducing TNF-α levels while maintaining or modulating IFN-γ expression. This balance could be particularly crucial for vaccine formulations, where reducing inflammation can mitigate potential adverse effects, and preserving IFN-γ ensures robust activation of antiviral and immune-regulatory pathways.

These findings pave the way for the development of adjuvant systems that can modulate inflammation while preserving immune activation, offering a more tailored approach to vaccine development. However, it is important to acknowledge the several limitations inherent in this study.

The first of limitations observed in this study is the small sample size and, consequently, the low number of experiments conducted. The use of PepTivator^®^ Influenza A H1N1 resulted in a poor i*n vitro* response from T cells, which limited the number of events and constrained our ability to effectively apply the stimulus for cytokine-producing T cell determination. A similar observation has been reported by other researchers applying the same stimulus, where cytokine production was suboptimal [36–39]. The low rate of cytokine-producing T cells could also be attributed to the use of EDTA, used as anticoagulant in the collected blood samples, which may have decreased T-cell activation by disrupting calcium-dependent signalling pathways, thereby diminishing the cells’ capacity to produce cytokines [40]. The production of cytokines by T-cells may also have been influenced by the use of frozen cells. Although freezing and thawing procedures were performed properly according to standardized methods, and the cultured cells exhibited high viability, the freezing process may still have caused subtle functional impairments that could affect cytokine production. A more detailed analysis using tetramers and/or stimulating antigen-specific T cells with inactivated viral particles would increase the identification of antigen-specific T cells and thus the number of the same detectable [21, 31]. Additionally, we were unable to distinguish between cells activated by prior encounters with the viral antigen and those resulting from immunization with the influenza vaccine after the recruitment [21].

Despite the limitations encountered in our work, our preliminary results lay the foundation for future research on OLE and BIRB 796, both individually and in combination, in the modulation of the immune response, particularly in older adults and following antigenic stimulation. However, further investigations are needed to fully explore their interesting potential.

## Conclusion

Our study examined the interplay between immunosenescence, inflammation, and influenza vaccine responses in older adults, with a particular focus on T cell immunophenotype and cytokine production. The small sample size and relatively low average age of the older adult group limited our ability to confirm specific responses and obtain statistically significant results, particularly with regard to the immunosenescence markers. Additionally, using a more targeted stimulus that specifically activates antigen-specific cells could have offered a clearer evaluation of the compounds’ effects. However, our investigation into the anti-inflammatory and antioxidant properties of OLE and BIRB 796 yielded promising results, suggesting their potential synergistic effect. Although the viral peptides used did not sufficiently stimulate cells in culture, our findings indicate that OLE and BIRB 796 may help regulate inflammation and oxidative stress associated with aging, potentially enhancing vaccine efficacy in older adults.

## Electronic supplementary material

Below is the link to the electronic supplementary material.

Supplementary Material 1:

Supplementary Material 2: Additional file 1 in dox format is uploaded with the manuscript. This file includes three sections: Material and Methods section with Protocol optimization; Results section with Results from preliminary tests; Additional Figures and Tables with an Additional Fig.  1 and an Additional Table 1.

## Data Availability

No datasets were generated or analysed during the current study.

## Abbreviations

PBMCs: Peripheral Blood Mononuclear Cells
OLE: Oleuropein
ROS: Reactive Oxygen Species
RNS: Reactive Nitrogen Species
NF-кB: Nuclear Factor kappa-light-chain-enhancer of activated B cells
p38 MAPK: p38 Mitogen-Activated Protein Kinases
ISOLDA: Improved vaccination Strategies for Older Adults
T0: Time 0
T1: Time 1
T2: Time 2
EDTA: Ethylene Diamine Tetraacetic Acid
SARS-CoV-2: Severe Acute Respiratory Syndrome COronaVirus 2
HBV: Hepatitis B Virus
HIV: Human Immunodeficiency Viruses
TCM: Central Memory T cells
TEM: CCR7-/CD45RA- Effector-Memory
TEMRA: Terminally differentiated CCR7-/CD45RA+
TNF-α: Tumor Necrosis Factor-α
IFN-γ: Interferon-γ
IL-10: Interleukin-10
PD1: Programmed Death 1
HA: Hemagglutinin protein
NP: Nucleocapsid Protein
MP1: Matrix Protein 1
H1N1: Influenza A virus subtype
PEPs: PepTivator^®^ Influenza A peptide pools
RFU: Relative Fluorescence Units
