# Supplementary figures and images for: Enhancing flu vaccine responses in older adults: preliminary insights from the ISOLDA study on immunosenescence and antioxidant and anti-inflammatory approaches
Source: Immun Ageing. 2025 Mar 26;22:13. doi: 10.1186/s12979-025-00506-y (PMC11938677; doi:10.1186/s12979-025-00506-y)

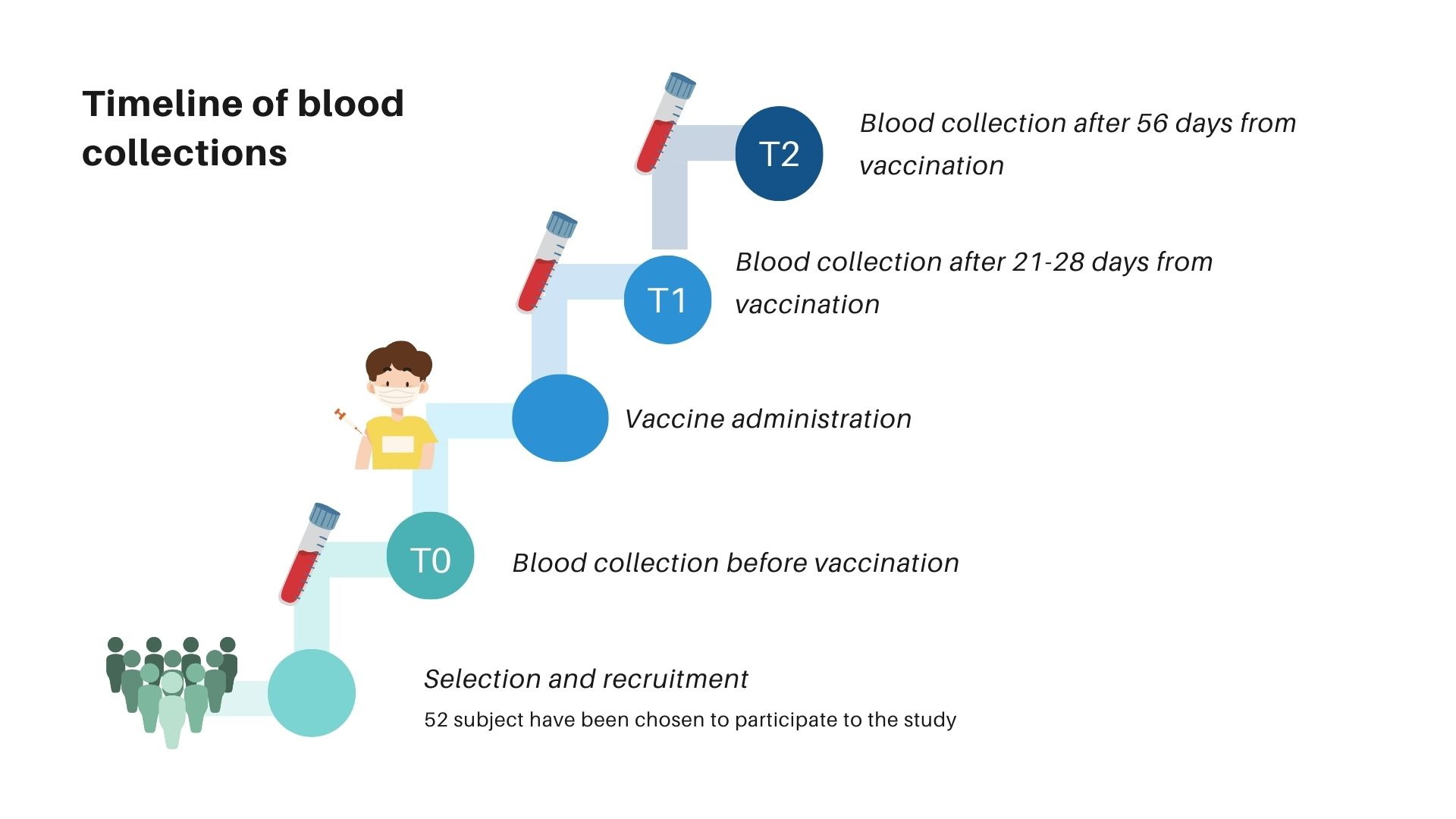

Supplement: Supplementary file 1 — Supplementary Material 1: [file 12979_2025_506_MOESM1_ESM.tiff]
