# Supplementary material for: Enhancing flu vaccine responses in older adults: preliminary insights from the ISOLDA study on immunosenescence and antioxidant and anti-inflammatory approaches
Source: Immun Ageing. 2025 Mar 26;22:13. doi: 10.1186/s12979-025-00506-y (PMC11938677; doi:10.1186/s12979-025-00506-y)
Supplement: Supplementary file 2 — Supplementary Material 2: Additional file 1 in dox format is uploaded with the manuscript. This file includes three sections: Material and Methods section with Protocol optimization; Results section with Results from preliminary tests; Additional Figures and Tables with an Additional Fig. 1 and an Additional Table 1. [file 12979_2025_506_MOESM2_ESM.docx]

**Additional File 1**

1. **Material and methods**

**1.1 Protocol optimisation**

To define and optimise the T cell stimulation protocol, we first performed preliminary experiments on Peripheral Blood Mononuclear Cells (PBMCs) from 3 young and 3 older donors at each time of recruitment (time zero, T0, time 1, T1, and time 2, T2). Thus, PBMCs were thawed, washed, counted using an automatic cell counter, and resuspended in a complete RPMI 1640 medium containing 5% human serum. They were plated at a density of 5×10^5^/well and 1×10^6^/well in a U-bottom 96-well plate and then incubated for 2 hours in controlled conditions (37°C and 5% CO_2_). At the end of incubation, PBMCs were treated with two different concentrations of OLE (5µM, 10µM) or pre-treated with them for 30 minutes at 37 °C with 5% CO_2_. The stimulation was conducted using PepTivator® Influenza A peptide pools specific for T cell activation (PepTivator® Influenza A nucleocapsid protein, matrix protein 1 and hemagglutinin protein, Miltenyi Biotec) at the concentration suggested by the manufacturer (0.6nmol of each peptide/mL), mixed and alone, for 4.5, 16, 24, 48, and 72 hours in the presence of different concentrations of BIRB 796 (80nM, 200nM, 800nM), at 37 °C and 5% CO_2_. PBMCs incubated for 72 hours were supplemented once with 40 U/mL of recombinant (rh) IL-2 (Roche) for maintenance. A negative control, represented by RPMI alone, was included for each experiment. As a positive control, we tested Lipopolysaccharide (LPS) from *E. coli* O55:B5 (Sigma-Aldrich), a highly immunogenic antigen, and CytoStim™ (Miltenyi Biotec), an artificial superantigen specific for stimulation of human effector/memory T cell. After 16, 24, 48, and 72 hours of incubation, cell viability was checked and cell supernatants were collected and frozen at -80°C until ELISA and ROS analysis. A simplified overview of the different culture conditions tested, for each patient and each recruitment is shown in Supplementary Table 1.

**1.2 Measurement of cytokines by ELISA**

Cell culture supernatants from preliminary tests were thawed and centrifuged for 20 minutes at 1.000×g. Interferon (IFN)-γ, interleukin (IL)-10, and tumor necrosis factor (TNF)-α released in the cell culture supernatant by T cells stimulated with PepTivator® Influenza A peptide pools and treated with OLE and BIRB 796 at the concentration indicated above, were measured by high sensitivity and specificity ELISA test, according to the manufacturer’s instruction (Cloud-clone Corp.).

**2** **Results**

**2.1 Results from preliminary tests**

The preliminary experiments indicated that a cell concentration of 1x10^6^ cells/well was suitable for optimal stimulation and cell viability definition (Supplementary Table 1). Additionally, rhIL-2 did not enhance the viability of PBMCs after 72 hours of stimulation. Indeed, the percentages of live cells at 4.5, 16, and 24 hours were similar to those at 72 hours of stimulation with rhIL-2, with live cell percentages of 80% and 77%, respectively. The 48 hours’ stimulation resulted in a live cell percentage of 65%, allowing it to be definitively excluded.

The stimulation for 16 hours of 1x10^6^ cells/well with a single peptide of the PepTivator® pools, resulted more efficiently than the stimulation with the PepTivator® mix in terms of cytokine release and cell viability. Indeed, ELISA assays performed showed a trend in the release of the cytokines analyzed, measured in optical density (OD), after overnight stimulation (16 hours), but not at 4.5, 24, 48, and 72 hours when the OD values were very low and under the limit of detection, given by the OD of the standards.

Data on viability, cytokine production release, and incubation with PepTivator® led to the definitive choice of cell stimulation time of 16 hours.

BIRB 796 at the concentration of 800nM showed a stronger effect on the cytokines OD according to the number of cells seeded (1x10^6^ cells/well). OLE concentrations evaluated (5µM and 10µM) determined a mild effect on the cell culture analyzed. In terms of OD trend, when added alone to the PBMCs, the major effect has been seen for OLE 10µM. Finally, the combination of OLE 10µM and BIRB 796 800nM gave better results.

The recruitment time T2 has been excluded from the later experiments because of the scarce stimulation, and thus undetectable levels of cytokines OD presumably due to the poorer presence of circulating memory T cells responsive to viral antigen after 56 days from the vaccination. After comparisons with LPS and CytoStim™, this last was chosen as a positive control because of the better results according to the release of the cytokine in the surnatant, in terms of OD and for the more specific stimulus applied.

Given the obtained results to evaluate the pro- and anti-inflammatory cytokines produced following influenza peptide stimulation and the treatment with the compounds under study, it was decided to use flow cytometry in the place of the ELISA test as the main method of analysis, which allowed us to detect with greater sensitivity and specificity pro- and anti-inflammatory cytokines released by T cells.

**3. Additional Figures and Tables**

**3.1 Additional Figure**

**Additional Figure 1.** History of blood sampling for enrolled patients. The figure describes the process of blood sample collection, from recruitment at time T0, before vaccine administration, 21-28 days after vaccination (T1), and 56 days after vaccination (T2).

**3.2 Additional Table**

**Additional Table 1**. Overview of the different conditions tested in the preliminary experiments.

|  |  | **1^st^ condition** | **2^nd^ condition** | **3^rd^ condition** |  |
| --- | --- | --- | --- | --- | --- |
|  | **T cells** | **+OLE**  **(5µM, 10µM)** | **+BIRB 796**  **(80nM, 200nM, 800nM)** | **+OLE+BIRB 796**  **(5µM+80nM; 10µM+80nM; 10µM+200nM; 10µM+800nM)** | **Incubation time** |
| Blank | No stimulation | ✗ | ✗ | ✗ | 4.5h, 16h, 24h, 48h and 72h |
| Antigenic stimulation  (0.6 nmol peptide/mL) | HA | ✓ | ✓ | ✓ |  |
|  | NP | ✓ | ✓ | ✓ |  |
|  | MP1 | ✓ | ✓ | ✓ |  |
|  | Mix of HA, NP, MP1 | ✓ | ✓ | ✓ |  |
| Positive control | LPS (1 µg/mL) | ✗ | ✗ | ✗ |  |
|  | CytoStim™ (20 μL/mL) | ✗ | ✗ | ✗ |  |
